# Appropriate sevoflurane concentration to stabilize autonomic activity during intubation with rocuronium in infants: a randomized controlled trial

**DOI:** 10.1186/s12871-015-0047-3

**Published:** 2015-04-29

**Authors:** Hiroshi Hanamoto, Aiji Boku, Yoshinari Morimoto, Mitsutaka Sugimura, Chiho Kudo, Hitoshi Niwa

**Affiliations:** 1 Department of Dental Anesthesiology, Osaka University Graduate School of Dentistry, 1-8, Yamadaoka, Suita, Osaka 565-0871 Japan; 2 Department of Anesthesiology, Graduate School of Dentistry, Kanagawa Dental University, 82, Inaoka-Cho, Yokosuka, Kanagawa 238-8580 Japan

**Keywords:** Intubation, Heart rate variability, Sevoflurane, General anesthesia, Infants, Autonomic nervous activity

## Abstract

**Background:**

In infants, sevoflurane is commonly used for induction of anesthesia, following which a muscle relaxant is administered to facilitate tracheal intubation. When rocuronium is used as the muscle relaxant, intubation may be performed before reaching an adequate depth of anesthesia because of its rapid onset. The purpose of this study was to investigate the optimal sevoflurane concentration that would minimize the impact of intubation on hemodynamics and autonomic nervous system (ANS) activity in infants.

**Methods:**

Sixty-one infants aged 1–6 months, undergoing cleft lip repair, were enrolled. Patients were randomly assigned to three end-tidal sevoflurane concentration (E’_Sevo_) groups, 3%, 4% and 5%. Anesthesia was induced with 5% sevoflurane with 100% oxygen, and rocuronium (0.6 mg/kg) was administered. The concentration of sevoflurane was adjusted to the predetermined concentration in each group. Mechanical pressure control ventilation via a face mask was commenced. Five minutes after E’_Sevo_ became stable at the predetermined concentration, tracheal intubation was performed. Immediately after tracheal intubation, ventilation was restarted at the same ventilator settings and continued for 150 seconds. Heart rate (HR) and mean arterial pressure (MAP) were measured 5 times in the 150 seconds after intubation. Normalized units (nu) of high frequency (HF: 0.04-0.15 Hz) and the ratio of low frequency (LF: 0.15-0.4 Hz) to HF components (LF/HF) of HR variability were calculated by MemCalc/Tonam2C™. Normalized units of HF (HFnu) and LF/HF reflect cardiac parasympathetic and sympathetic activity, respectively.

**Results:**

After intubation, HR increased slightly in all groups and MAP increased by 9.2% in the E’_Sevo_-3% group. LF/HF increased (p < 0.01) and HFnu decreased (p < 0.01) in all groups 30 seconds after intubation. HFnu was lower (p < 0.001) and LF/HF was higher (p = 0.007) in the E’_Sevo_-3% group than in E’_Sevo_-5% group. ANS responses to intubation were reduced in a dose-dependent manner.

**Conclusions:**

Sympathomimetic and parasympatholytic responses to intubation in the E’_Sevo_-3% group were much greater than those in the E’_Sevo_-5% group. During tracheal intubation in infants, 4% or 5% sevoflurane is appropriate for prevention of sympathetic hyperactivation and maintenance of ANS balance as compared to 3% sevoflurane, when a muscle relaxant is co-administered.

**Trial registration:**

The study was registered at UMIN-CTR (UMIN000009933).

## Background

In infants, sevoflurane is commonly used for inhalational induction of anesthesia and tracheal intubation without muscle relaxants is well accepted [1]. However, muscle relaxants are widely used to facilitate tracheal intubation even in infants, because of the potential risk for movement of the vocal cords, coughing and laryngospasm [2,3]. Moreover, avoidance of muscle relaxants may increase the risk of difficult tracheal intubation [4]. In addition, there is no reason to withhold use of muscle relaxants, such as rocuronium, even for short duration surgeries, due to the ease of sugammadex-induced reversal of relaxant activity even with short procedures [5,6]. On the other hand, muscle relaxants can allow tracheal intubation even under light depths of sevoflurane anesthesia [7]. In particular, the onset time of rocuronium is so rapid that an adequate depth of anesthesia for intubation may not yet have been achieved.

Although several studies have assessed sevoflurane concentration during tracheal intubation without muscle relaxants in children, the optimal sevoflurane concentration for intubation in infants when using muscle relaxants is unknown. We hypothesized that laryngoscopy or intubation under a light depth of sevoflurane anesthesia might affect the autonomic nervous system (ANS), despite excellent intubating conditions. Although muscle relaxants facilitate tracheal intubation, their use may result in intubation under a lighter depth of sevoflurane anesthesia, which would induce sympathetic nervous system activation and the possibility of hemodynamic instability. Therefore, appropriate anesthetic depth during intubation is recommended to prevent ANS imbalance. ANS activity can be evaluated by measuring heart rate variability (HRV) indices. Spectral analysis of HRV is widely used as a non-invasive method to assess cardiac sympathetic and parasympathetic function [8,9]. The purpose of this study was to investigate the appropriate sevoflurane concentration that would minimize changes in heart rate, heart rate variability and non-invasive blood pressure during tracheal intubation in infants.

## Methods

### Study design

The study protocol was approved in November 2010 by the Institutional Review Board and Ethics Committee of Osaka University Dental Hospital (ref: H22-E22-1), and was registered with UMIN-CTR (UMIN000009933). Written informed consent was obtained from the parents or guardians of all the subjects. We studied 68 cleft lip patients aged between 1 and 6 months old, with an American Society of Anesthesiologists physical status of 1 or 2, who were undergoing general anesthesia for elective cheiloplasty from January 2011 to October 2013. A history of epilepsy or significant cardiopulmonary, renal or hepatic dysfunction, symptoms of an upper respiratory tract infection, or predictive signs of difficult intubation were considered criteria for exclusion from the study.

### Study protocol

Patients were randomly allocated in a single-blinded fashion (patients and parents were blinded) to three groups of end-tidal sevoflurane concentrations (E’_Sevo_) of 3%, 4%, and 5%, using computer-generated randomized numbers without any restriction before the induction of anesthesia. All patients were made to fast for 5 hours before the induction of anesthesia and did not receive premedication. After arrival at the operating room, heart rate (HR), electrocardiogram, pulse oximetry (SpO_2_) and noninvasive mean arterial blood pressure (MAP) were monitored (IntelliVue MP50, Philips, Amsterdam, the Netherlands).

Inhalation anesthesia was induced by a senior anesthetist via a face mask, with 5% sevoflurane with 100% oxygen at a fresh gas flow of 6 l/min using a semi-closed system, with monitoring of end-tidal carbon dioxide (E’_CO2_) and E’_Sevo_. After venous access was obtained, 0.6 mg/kg rocuronium (Eslax, MSD, Tokyo, Japan) was administered. After cessation of spontaneous respiration, the concentration of sevoflurane was adjusted to the predetermined concentration in each group. Mechanical pressure control ventilation via a face mask was commenced. Since the respiratory condition might influence HRV, we applied the same ventilator settings before and after intubation. In our preliminary study, we determined the ventilator settings required to achieve an E’_CO2_ partial pressure of 35–40 mmHg after intubation as: peak pressure, 13 cm H_2_O; respiratory rate, 18 breaths/min; and inspiratory/expiratory ratio, 1:2.5 (Fabius Tiro, Dräger, Lübeck, Germany). After reaching the predetermined value, the E’_Sevo_ was kept constant, and the ratio of predetermined end-tidal to inspiratory sevoflurane concentration was maintained at 0.90-1.00 for 5 minutes. Five minutes after E’_Sevo_ became stable at the predetermined concentration, we recorded control data. The trachea was intubated with an uncuffed, 3.5 mm ID RAE™ pediatric orotracheal tube (Mallinckrodt, Dublin, Ireland) by a trained anesthetist (H.H.). Tracheal intubation was performed using a laryngoscope (HEINE Classic + ™ Macintosh Fiber Optic Blade, size 1; HEINE, Herrsching, Germany) and the tip of the blade was placed in the epiglottic vallecula. Immediately after tracheal intubation, mechanical pressure controlled ventilation was restarted at the same ventilator settings.

If a leak occurred at an inflation pressure less than 8 cm H_2_O and tidal volume was less than 3 ml/kg, the tracheal tube was exchanged for one that was 0.5 mm larger, and the infant was excluded from analysis. If tracheal intubation was difficult and repeated laryngoscopy was needed, data collection was discontinued.

### Data collection methods

The fast peaks of the R-R interval (RRI) were continuously monitored. Data were transferred to a computer loaded with heart rate variability analysis software (MemCalc/Tonam2C™, GMS, Tokyo, Japan). RRI data were obtained at 1-ms sampling intervals and analyzed by the maximum entropy method at high resolution with the “MemCalc” computer program. “MemCalc” executes a linearized version of the nonlinear least squares method for fitting analysis in the domain, combined with the maximum entropy method or spectral analysis method in the frequency domain [10]. It provides reliable analysis of HRV over a minimum interval of 30 seconds and recognizes the abnormal RRI of premature beats or artifacts, including noise, and removes it automatically. The power of the RRI (ms^2^) with LF (0.04-0.15 Hz) and HF (0.15-0.4 Hz) bands was calculated. The total power (TP, 0.04-0.5 Hz) was obtained by addition of LF and HF. To confirm normal distribution, HF (proportional power) was calculated as a percentage of TP (normalized unit of HF (HFnu) = HF / TP × 100), and the LF/HF ratio was also assessed.

### Study variables

In this study, the outcome variables measured were HR, MAP, HFnu and LF/HF. These outcome variables, except for MAP, were measured at six time points: T0, before intubation (5 minutes after stable state); T1, 30 seconds; T2, 60 seconds; T3, 90 seconds; T4, 120 seconds; and T5, 150 seconds after tracheal intubation (at resumption of ventilation). MAP was measured at four time points: T0, T1, T3 and T5. The other variables studied were demographic and perioperative variables. Demographic variables included age and body weight, while perioperative measurements included intubation time, tidal volume, E’_CO2_ and E’_Sevo_. Intubation time was defined as the time from the start of laryngoscopy to the resumption of ventilation.

### Statistical analysis

Estimated sample size was calculated based on the results of a pilot study (n = 15), where average LF/HF was 2.8 with a standard deviation (SD) of 4.5. A difference of 2.0 in the LF/HF between the E’_Sevo_-3% and E’_Sevo_-5% groups, 30 seconds after tracheal intubation, was considered clinically relevant. With a type-2 error of 20% at a two-sided 5% significance level, we estimated that 54 patients had to be included in this study. Finally, 68 patients were required in total when considering a dropout rate of 20%.

Statistical analysis was performed with the SPSS Statistics version 22.0 software package (IBM, Armonk, NY). Age, body weight and respiratory parameters were expressed as mean ± SD and compared by one-way analysis of variance (ANOVA). Sex was expressed as number of patients and analyzed statistically by the Chi-square test. HR, MAP, HFnu and LF/HF were expressed as mean ± standard errors, and compared over time by one-way ANOVA for repeated measures, followed by the Tukey test to adjust for multiple comparisons, where appropriate. Statistical comparisons between groups were assessed using one-way ANOVA followed by the Tukey test to adjust for multiple comparisons, where appropriate. A p-value of <0.05 was considered significant.

## Results

**Figure 1:**
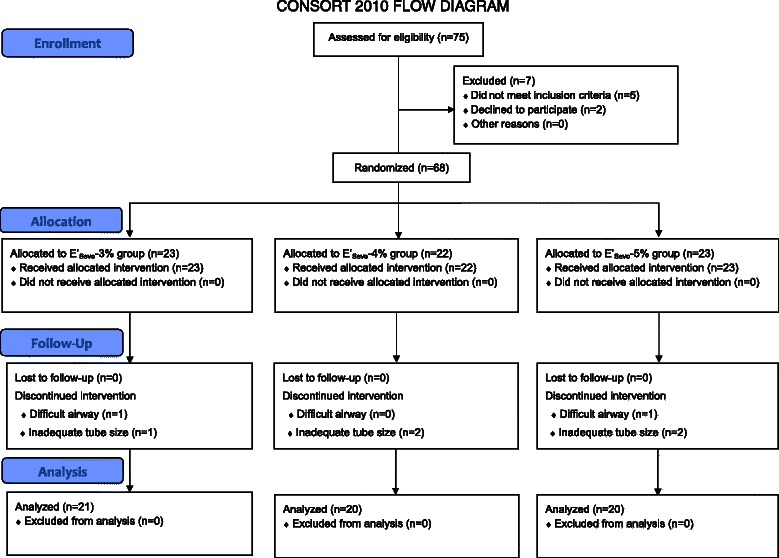
CONSORT flow diagram.

The flow diagram of the conduct of the study is shown in Figure 1. Of the 75 patients who were initially assessed, 68 patients were randomly allocated to the three groups, and 61 patients successfully completed the study without any complications. There were no significant differences in demographic data and respiratory parameters among the three groups (Table 1). The E’_Sevo_ of the three groups was 2.91 ± 0.11% (E’_Sevo_-3% group), 3.88 ± 0.17% (E’_Sevo_-4% group) and 4.82 ± 0.24% (E’_Sevo_-5% group).

**Table 1:** **Demographic data and respiratory parameters**

Variables	E’_Sevo_-3%	E’_Sevo_-4%	E’_Sevo_-5%	P value
	(n = 21)	(n = 20)	(n = 20)	
Age (days)	95.2 ± 19.2	90.0 ± 13.4	96.7 ± 14.9	0.382
Sex (male/female)	14/7	10/10	12/8	0.552
Body weight (kg)	5.9 ± 0.9	5.8 ± 0.9	6.2 ± 0.7	0.303
Intubation time (sec)	29.8 ± 10.4	30.9 ± 9.5	28.0 ± 7.4	0.598
Tidal volume (ml)				
Before intubation (T0)	79.1 ± 16.9	79.1 ± 21.1	80.4 ± 19.4	0.971
After intubation (T1)	53.1 ± 15.5	46.9 ± 17.2	51.5 ± 16.1	0.456
E’_CO2_ (mmHg)				
Before intubation (T0)	27.5 ± 4.4	26.4 ± 3.1	28.8 ± 3.9	0.147
After intubation (T1)	35.9 ± 4.3	37.1 ± 6.1	37.3 ± 4.3	0.624

Demographic data and respiratory parameters in each group. Data are expressed as mean ± SD or number of patients.

E’_CO2_, end-tidal carbon dioxide.

**Figure 2:**
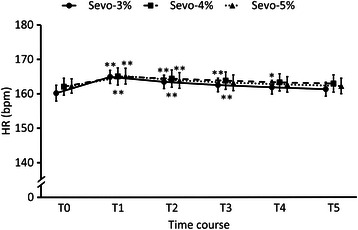
Time course of heart rate. Time course of heart rate (HR) before and after intubation in E’_Sevo_-3% (circle points and solid line), E’_Sevo_-4% (square points and broken line) and E’_Sevo_-5% (triangle points and dotted line) groups. Data are presented as mean (standard error). T0, before intubation; T1, 30 seconds; T2, 60 seconds; T3, 90 seconds; T4, 120 seconds; and T5, 150 seconds after tracheal intubation. *, p < 0.05 vs. T0; **, p < 0.01 vs. T0; †, p < 0.05 E’_Sevo_-3% vs. E’_Sevo_-5%.

**Figure 3:**
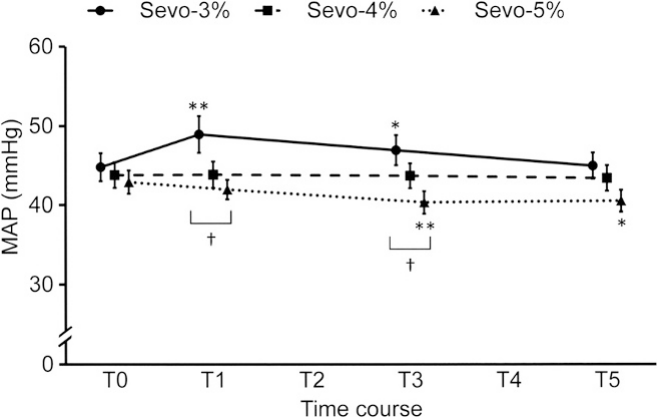
Time course of mean arterial pressure. Time course of mean arterial pressure (MAP) before and after intubation in E’_Sevo_-3% (circle points and solid line), E’_Sevo_-4% (square points and broken line) and E’_Sevo_-5% (triangle points and dotted line) groups. Data are presented as mean (standard error). T0, before intubation; T1, 30 seconds; T2, 60 seconds; T3, 90 seconds; T4, 120 seconds; and T5, 150 seconds after tracheal intubation. **, p < 0.01 vs. T0; †, p < 0.05 E’_Sevo_-3% vs. E’_Sevo_-4%; ††, p < 0.01 E’_Sevo_-3% vs. E’_Sevo_-5%.

**Figure 4:**
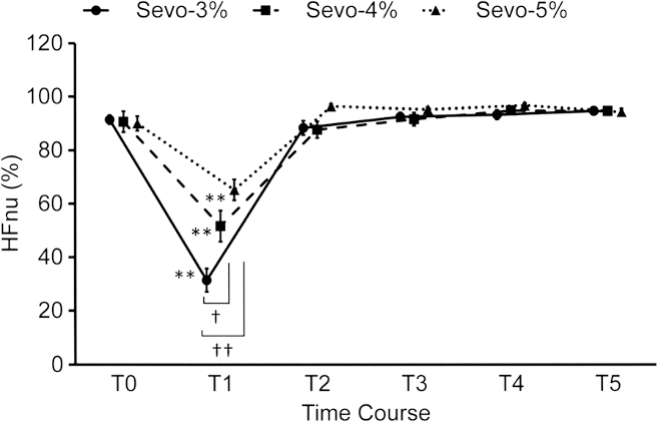
Time course of normalized units of HF. Time course of normalized units of HF (HFnu) before and after intubation in E’_Sevo_-3% (circle points and solid line), E’_Sevo_-4% (square points and broken line) and E’_Sevo_-5% (triangle points and dotted line) groups. Data are presented as mean (standard error). T0, before intubation; T1, 30 seconds; T2, 60 seconds; T3, 90 seconds; T4, 120 seconds; and T5, 150 seconds after tracheal intubation. **, p < 0.01 vs. T0; †, p < 0.05 E’_Sevo_-3% vs. E’_Sevo_-4%; ††, p < 0.01 E’_Sevo_-3% vs. E’_Sevo_-5%.

**Figure 5:**
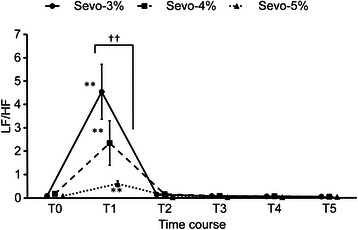
Time course of low frequency to high frequency components. Time course of low frequency to high frequency components (LF/HF) before and after intubation in E’_Sevo_-3% (circle points and solid line), E’_Sevo_-4% (square points and broken line) and E’_Sevo_-5% (triangle points and dotted line) groups. Data are presented as mean (standard error). T0, before intubation; T1, 30 seconds; T2, 60 seconds; T3, 90 seconds; T4, 120 seconds; and T5, 150 seconds after tracheal intubation. **, p < 0.01 vs. T0; ††, p < 0.01 E’_Sevo_-3% vs. E’_Sevo_-5%.

Changes in each parameter before and after tracheal intubation are indicated in Figures 2, 3, 4 and 5. HR increased in all groups at T1 and T2. HR increased maximally at T1 by 2.9% (p < 0.001) in the E’_Sevo_-3% group, by 1.9% (p < 0.001) in the E’_Sevo_-4% group and by 1.7% (p < 0.001) in the E’_Sevo_-5% group. There were no significant differences in HR among groups at each time point (Figure 2). MAP increased by 9.2% (p < 0.001) at T1 and by 4.8% (p = 0.04) at T3 in the E’_Sevo_-3% group. MAP decreased by 5.3% (p = 0.006) at T3 and by 4.9% (p = 0.011) at T5 in the E’_Sevo_-5% group. MAP was higher in the E’_Sevo_-3% group than in the E’_Sevo_-5% group at T1 (p = 0.034) and T3 (p = 0.025) (Figure 3). LF/HF increased and HFnu decreased significantly at T1 in all groups. HFnu was lower in the E’_Sevo_-3% group than in the E’_Sevo_-4% group (p = 0.011) and in the E’_Sevo_-5% group (p < 0.001) at T1. LF/HF was higher (p = 0.007) in the E’_Sevo_-3% group than in the E’_Sevo_-5% group at T1. ANS responses to intubation were reduced in a dose-dependent manner (Figures 4 and 5).

## Discussion

The major findings of this study were that (1) hemodynamic changes due to intubation were within 10% of pre-intubation values, (2) sympathetic activation was observed just after intubation, and (3) these responses were smaller in the E’_Sevo_-5% group than in the E’_Sevo_-3% group.

In general, ANS activity cannot be measured directly and continuously in humans. Assessment of heart rate variability noninvasively provides information about several useful indices that are associated with ANS activity. HF (HFnu) is associated with cardiac parasympathetic activity [7]. The LF/HF ratio is considered to represent sympathovagal balance or the effect of modulation of sympathetic activity [8]. During general anesthesia, although analysis of the absolute value of HF may not be appropriate for interpreting changes in the ANS because of overall depression of the TP, the relative value (HFnu) may still be useful for interpretation of ANS activity when an overall decrease in all components occurs [11]. Therefore, we used the relative value in our study.

In the present study, we estimated that increase in sympathetic activity and decrease in parasympathetic activity occurred 30 seconds after intubation, as indicated by the increase in LF/HF and decrease in HFnu, respectively. The increase in LF/HF and decrease in HFnu were smaller in the E’_Sevo_-5% group than in the E’_Sevo_-3% group. Therefore, we estimated that the ANS responses to intubation were reduced by sevoflurane in a dose-dependent manner.

It is important to consider the respiratory conditions that have an impact on HRV [12]. As long as respiratory rate remains unchanged, changes in end-tidal CO_2_ or tidal volume have little impact on HRV in anesthetized patients [13]. However, because apnea is inevitable during laryngoscopy and tracheal intubation, it is meaningless to compare the results of HRV obtained during these manipulations with those during mechanical ventilation [14]. Therefore, in this study, in order to avoid the influence of apnea, HRV data from the start of laryngoscopy until 30 seconds after the restart of ventilation were excluded, since HRV data are calculated from the RRIs for 30 seconds in the MemCalc™ system.

In the present study, we investigated the appropriate depth of sevoflurane anesthesia for intubation in infants. Although minimum alveolar concentration (MAC) [15] is usually determined by the up-and-down method, we could not use this method because HRV is usually assessed using relative changes, and the cutoff value of ANS activity is unclear. Therefore, we compared the hemodynamic and ANS changes induced by intubation at different sevoflurane concentrations. The concentration of sevoflurane was determined with reference to the value of MAC for tracheal intubation (MAC-TI) or blocking adrenergic responses (MAC-BAR) in infants.

The MAC in pediatric patients changes with age [16-19]. The MAC of sevoflurane is highest in younger patients: 3.3% for neonates, 3.2% for infants 1 to 6 months old and 2.5% for children older than 6 months [16,17]. MAC-TI is assessed based on the intubating conditions, such as coughing, movements of the vocal cords or limbs, and whether tracheal intubation is smooth or not [16,18,20-22]. According to previous studies, the MAC-TI of sevoflurane at different ages was 2.69% (age, 1–9 years), 3.10% (age, 2–8 years) and 2.49% (age, 2–6 years). However, there was no available data for infants less than 1 year of age. The ratio of MAC-TI/MAC is reported to be 1.33 in children (age, 1–9 years) [18]. If this ratio is applied to our patients (aged 1–6 months), the MAC-TI is calculated as 4.26%. Clinically, when intubating with sevoflurane alone, a higher concentration than MAC-TI is required to accomplish successful intubation. Hence, anesthetists need to refer to the 95% effective dose of MAC-TI (MAC-TI_ED95_) rather than MAC-TI, to achieve effective reduction of the stress associated with intubation in almost all patients. Although the MAC-TI_ED95_ in children (age, 1–7 years) was reported as 3.54%, [16] its value in infants is unknown. In the present study, considering MAC-TI and MAC-BAR, the ANS and hemodynamic responses to intubation were explored at 3%, 4%, and 5% sevoflurane anesthesia.

In clinical settings, muscle relaxants are commonly used to facilitate tracheal intubation even in pediatric patients [1]. With the use of muscle relaxants, tracheal intubation can be performed even under light or moderate anesthesia [7,23]. However, when using muscle relaxants, it is difficult to evaluate the optimal depth of anesthesia for intubation because muscle relaxants can improve intubation conditions regardless of the depth of anesthesia. Therefore, other measurements are required to determine the optimal depth of anesthesia.

MAC-BAR is a clinically important index that is assessed by the increase in HR or blood pressure. The cut-off value for determination of MAC-BAR is usually an increase in HR or MBP by 10% [24] to 15% [25-27]. In the present study, the changes in HR and MAP were within 10% after intubation, which is considered to be acceptable. On the other hand, ANS responses are extremely sensitive to noxious stimulation even if hemodynamic changes are small. Sympathetic activation due to intubation was observed in all groups and was larger in the E’_Sevo_-3% than in the E’_Sevo_-5% group. Although MAP remained unchanged in the E’_Sevo_-4% group, 5% sevoflurane reduced it. These results suggest that 5% sevoflurane might be too high for intubation. Therefore, 4% sevoflurane, which would minimize the changes in ANS activity without producing side effects, such as hemodynamic depression, might be adequate for intubation. In our study, although we kept the sevoflurane concentration constant until 150 seconds after tracheal intubation to conduct the analyses, inhalation of 5% sevoflurane until tracheal intubation, with a decrease in the sevoflurane dose to a level required for maintenance of anesthesia immediately after tracheal intubation, might also be acceptable in daily clinical practice.

Measurement of plasma catecholamine concentration is one of the most common methods for assessing ANS activity. Although the catecholamine response to tracheal intubation is similar to heart rate and blood pressure responses in children, the magnitude of the changes seems to be different [28]. Evaluation of the ANS response to intubation by catecholamine measurements is difficult because it is not sensitive enough and requires repeated blood sampling in a short time interval. Therefore, we used HRV to assess the ANS response in this study, because HRV data is available non-invasively every 30 seconds by using the MemCalc system.

In our study, only sevoflurane was used as the anesthetic agent during induction of anesthesia and tracheal intubation. This method makes it possible to investigate the effect of sevoflurane itself. However, sevoflurane with fentanyl [21], remifentanil or propofol [29] can also be used for tracheal intubation in children. Further study on the effects of these adjuvant drugs combined with sevoflurane on heart rate variability is needed.

There are some limitations to the present study. First, the conventional method of determining MAC-TI requires a constant end-tidal concentration of volatile anesthetics to be maintained for at least 15 minutes, to establish equilibration between cerebral, arterial blood and alveolar gas tensions before tracheal intubation [18]. However, since administration of inhalational anesthetics for 15 minutes in order to achieve the steady state is not followed in routine clinical practice, we did not wait for 15 minutes before obtaining measurements in our patients as it would mean an excessive exposure to anesthetics purely for research purposes, which we believe is unethical. Although Inomata et al. studied MAC-TI without achievement of the steady state (rapid method) [22], we considered 5 minutes adequate for achieving the steady state, in accordance with their other recent study on MAC-TI [21]. Second, in this study, pre-intubation E’_CO2_ was smaller than post-intubation E’_CO2_. Although the ventilator settings were equal before and after intubation, the difference in E’_CO2_ was considered to be acceptable because of the changes in dead space before and after tracheal intubation. Similar E’_CO2_ data were shown in a previous study [30]. Third, the optimal anesthetic depth during tracheal intubation is still an issue. Tracheal intubation should be performed principally for the benefit of the patient rather than the satisfaction of the anesthesiologist [6]. The target anesthetic depth is different for each anesthesiologist and the ideal target, whether prevention of the ANS response, hemodynamic response, body movement or patients’ memory, is still controversial. Further studies and discussion about optimal anesthetic depth during tracheal intubation are still needed.

## Conclusion

During tracheal intubation in infants, 4 or 5% of sevoflurane is appropriate for the prevention of sympathetic hyperactivation and maintenance of ANS balance as compared to 3% sevoflurane, when a muscle relaxant is co-administered.

## Abbreviations

ANS: Autonomic nervous system
E’_Sevo_: End-tidal sevoflurane concentration
HR: Heart rate
MAP: Mean arterial pressure
nu: Normalized units
HF: High frequency
LF: Low frequency
LF/HF: Low frequency to high frequency components
HFnu: Normalized unit of HF
HRV: Heart rate variability
SpO_2_: Pulse oximetry
E’_CO2_: End-tidal carbon dioxide
RRI: R-R interval
TP: Total power
SD: Standard deviation
ANOVA: Analysis of variance
MAC: Minimum alveolar concentration
MAC-TI: Minimum alveolar concentration for tracheal intubation
MAC-BAR: Minimum alveolar concentration for blocking adrenergic responses
